# Biogenic Synthesis of Silver Nanoparticles Mediated by *Aronia melanocarpa* and Their Biological Evaluation

**DOI:** 10.3390/life14091211

**Published:** 2024-09-23

**Authors:** Andreia Corciovă, Cornelia Mircea, Adrian Fifere, Ioana-Andreea Turin-Moleavin, Irina Roşca, Irina Macovei, Bianca Ivănescu, Ana-Maria Vlase, Monica Hăncianu, Ana Flavia Burlec

**Affiliations:** 1 Faculty of Pharmacy, “Grigore T. Popa” University of Medicine and Pharmacy, 16 University Street, 700115 Iasi, Romania; maria.corciova@umfiasi.ro (A.C.); cornelia.mircea@umfiasi.ro (C.M.); monica.hancianu@umfiasi.ro (M.H.); ana-flavia.l.burlec@umfiasi.ro (A.F.B.); 2 Centre of Advanced Research in Bionanoconjugates and Biopolymers Department, “Petru Poni” Institute of Macromolecular Chemistry, 41A Grigore Ghica Voda Alley, 700487 Iasi, Romania; fifere@icmpp.ro (A.F.); moleavin.ioana@icmpp.ro (I.-A.T.-M.); rosca.irina@icmpp.ro (I.R.); 3 Department of Pharmaceutical Botany, Faculty of Pharmacy, Iuliu Hațieganu University of Medicine and Pharmacy, 8 Victor Babeș Street, 400012 Cluj-Napoca, Romania; gheldiu.ana@umfcluj.ro

**Keywords:** silver nanoparticles, *Aronia melanocarpa*, green synthesis, physicochemical characterization, phytotoxicity, antimicrobial activity, antioxidant activity

## Abstract

In the present study, two *A. melanocarpa* berry extracts were used for the synthesis of silver nanoparticles (AgNPs). After the optimization of synthesis, the AgNPs were characterized using UV–Vis, FTIR, EDX, DLS, and STEM analyses. The stability in different media, phytotoxicity, as well as antimicrobial and antioxidant activities were also evaluated. The ideal synthesis conditions were represented by a 3 mM AgNO_3_ concentration, 1:9 extract:AgNO_3_ volume ratio, alkaline medium, and stirring at 40 °C for 120 min. The synthesis was confirmed by the surface plasmon resonance (SPR) peak at 403 nm, and the strong signal at 3 keV from the EDX spectra. FTIR analysis indicated that polyphenols, polysaccharides, and amino acids could be the compounds responsible for synthesis. Stability tests and the negative zeta potential values showed that phytocompounds also play a role in the stabilization and capping of AgNPs. The preliminary phytotoxicity studies on *T. aestivum* showed that both the extracts and their corresponding AgNPs had an impact on the growth of roots and shoots as well as on the microscopic structure of leaves. The synthesized AgNPs presented antimicrobial activity against *S. aureus*, *E. coli*, and *C. albicans*. Moreover, considering the results obtained in the lipoxygenase inhibition, the DPPH and hydroxyl scavenging activities, and the ferrous ion chelating assay, AgNPs exhibit promising antioxidant activity.

## 1. Introduction

Nanotechnology is an advanced field of research that is based on the use of nano-sized materials in different areas of interest (medical, environmental, industrial, and agricultural). From this field of study emerges nanobiotechnology, which aims to overcome the drawbacks of conventional biotechnological and nanoparticle synthesis techniques by using the size- and structure-dependent features of nanomaterials obtained using natural sources in order to create a sustainable future [1,2].

The classification of nanoparticles (NPs) generally includes organic (dendrimers, micelles, nanogels, polymeric, and protein NPs), inorganic (quantum dots, metal, metal oxide, and silica-based NPs), and carbon-based NPs [3,4]. Among these, metal NPs have been shown to have a wide range of applications in the medical field, including activities related to diagnosis (gold, cadmium sulfide, silver, palladium, iron-, copper-, zinc oxide, and titanium dioxide NPs), tumor targeting (silver, gold, platinum, cerium, copper, iron, nickel, magnesium, and zinc oxide NPs), angiogenesis (copper, gold, titanium, and silver NPs), antiviral (silver, gold, copper, zinc oxide, and iron-oxide NPs), anti-inflammatory (silver, gold, selenium, zinc oxide, and titanium dioxide NPs), or antimicrobial effects (silver, gold, zinc oxide, copper, and copper oxide NPs) [3,5,6,7,8,9].

For the synthesis of NPs, in addition to top-down methods (mechanical or ball milling, chemical etching, thermal or laser ablation, sputtering) and bottom-up methods (chemical or electrochemical precipitation, vapor deposition, atomic/molecular condensation, spray and laser pyrolysis, and aerosol processes), bioreduction, which implies the use of a whole organism/tissue or cell-free extracts from natural sources, can also be considered. Such natural sources include plants, yeasts, fungi, algae, actinomycetes, cyanobacteria, mushrooms, lichen, and seaweeds [10,11].

Plant-based NP synthesis represents an approach that provides an appropriate compatibility between the synthesized material and the environment in which it will be distributed. This method generates NPs with unique physicochemical properties, considering their size, shape, composition, magnetic, electrical, optical, photo-electrochemical, catalytic, and even biological characteristics. In addition, this synthesis process is energy-efficient, fast, secure, environmentally friendly, and cost-effective [12]. The advantages provided by phytocompounds that take part in biosynthesis and are present in the resulting nanosystems also contribute to the properties of NPs. The following classes of compounds are particularly useful in the production of NPs: polyphenols, flavonoids, saponins, alkaloids, amides, glycosides, tannins, and terpenes [13,14,15].

*Aronia melanocarpa* or black chokeberry is a perennial plant belonging to the *Rosaceae* family, native to North America, that is cultivated in Europe and Asia as well [16,17]. Black chokeberry fruits are currently used in health-promoting products such as juices, jams, wines, and teas. Since they are rich in phytocompounds, they also have various biological properties in addition to their potential as antioxidants [18]. Numerous studies demonstrated the antitumor [19,20,21,22], antimutagenic [23,24], cardioprotective [25,26,27], hepatoprotective [28,29], gastroprotective [30], antidiabetic [31,32], anti-inflammatory [33,34], immunomodulatory [35,36], antibacterial [37,38], antiviral [39,40], and radioprotective [18,41] activities of different types of preparations containing black chokeberry.

Despite the various biological activities of *A. melanocarpa*, its potential for metal NP production has not been thoroughly studied. To the best of our knowledge, there are only two papers on the green hydrothermal synthesis of *A. melanocarpa* extract-functionalized AuNPs, AgNPs, Au/AgNPs, and ZnO nanoclusters and their application for removing organic dyes (methylene blue or rhodamine) from water. Furthermore, the results cannot be directly compared since the applied synthesis conditions were different [42,43].

Potential applications of such phyto-functionalized AgNPs rely on their antimicrobial and antioxidant activities, which could be further exploited for nano-pesticides, food packaging, pest management, crops, and food protection as well as for biomedical applications (e.g., drug-delivery formulations, diagnosis agents, and tissue reconstruction) [3].

Consequently, our study focuses on the ecofriendly synthesis of silver nanoparticles (AgNPs) that use two *A. melanocarpa* berry extracts as reducing and capping agents. After optimization of the conditions, different methods were used to confirm the synthesis of AgNPs, such as UV–Vis spectroscopy, Fourier-transform infrared (FTIR) spectroscopy, energy-dispersive X-ray (EDX), dynamic light scattering (DLS), and scanning transmission electron microscope (STEM) analyses. The stability of AgNPs in different environments over time was also evaluated. A preliminary study on the phytotoxicity of AgNPs was carried out using the *Triticum aestivum* assay. Moreover, the antimicrobial and antioxidant activities of the synthesized AgNPs were investigated using various methods.

## 2. Materials and Methods

### 2.1. Extract Obtainment and Analysis

For AgNPs synthesis, two types of aqueous extracts were used. The first one was obtained directly from *A. melanocarpa* black chokeberries, while the second one was obtained from a lyophilized extract prepared in the Department of Pharmacognosy, Faculty of Pharmacy, “Grigore T. Popa” University of Medicine and Pharmacy, Iași, Romania, and put at our disposal.

For the first extract (E1), black chokeberries were purchased from a local natural organic products market in Iasi, Romania. The extract was prepared by adding 10 g of berries powder to 100 mL distilled water, followed by stirring for 30 min at 40 °C. The mixture was cooled at room temperature, filtered using Whatman filter paper no. 1, and the extract was further used for AgNPs synthesis. First, this extract was chemically characterized using an Agilent 1100 HPLC Series system (Agilent, Santa Clara, CA, USA) equipped with a degasser, binary gradient pump, column thermostat, autosampler, and UV detector. The HPLC system was coupled with an Agilent 1100 mass spectrometer (LC/MSD Ion Trap VL). For the separation, a reverse-phase analytical column was employed (Zorbax SB-C18 100 × 3.0 mm i.d., 3.5 μm particle, Agilent, Santa Clara, CA, USA); the temperature was set at 48 °C. The MS system operated using an electrospray ion source in negative mode. The phenolic compounds used as standards were caftaric, gentisic, caffeic, chlorogenic, 4-O-caffeoylquinic, *p*-coumaric, ferulic and sinapic acids, vitexin, hyperoside, vitexin 2-O-rhamnoside, isoquercitrin, rutoside, myricetin, fisetin, quercitrin, kaempferitrin, quercetin, kaempferol-3-rhamnoside, patuletin, luteolin, kaempferol, and apigenin [44,45,46]. A second validated LC-MS analytical method was used to identify six other polyphenols: epicatechin, catechin, syringic, gallic, protocatechuic, and vanillic acids. Chromatographic separation was performed using the same equipment and on the same analytical column mentioned above [47,48]. The identification and quantification of each bioactive compound from the analyzed samples was made as previously described [49]. The detection of bioactive compounds was performed in MS mode. For the identification of each bioactive compound from the samples, the MS spectra/traces were compared with library spectra. After MS identification, the UV trace was further used for the quantification of compounds. For the identified compounds, the calibration curve of their corresponding standards was considered for the quantification of their peak areas. For both methods, the results were expressed as μg bioactive compound per mL sample. Furthermore, the total phenolic content was determined using the previously described UV–Vis spectrophotometric Folin–Ciocalteu method. Gallic acid was used as standard and the results were expressed in mg gallic acid equivalents (GAE) per mL sample [50].

For the second extract (E2), a quantity of the lyophilized extract equivalent to 10 g of berry powder was used and then proceeded as in the case of E1. The lyophilized extract was previously characterized and contains selective fractions of anthocyanosides [51]. Since the first extract (E1) has a much more complex chemical composition and since the second extract (E2) was already previously characterized [51], the FTIR and chromatographic analyses were carried out only on E1, as additional studies.

### 2.2. Optimization of AgNPs Synthesis

The synthesis of AgNPs was monitored by recording the UV–Vis spectra of the reaction mixture in the 300–600 nm range, using different parameters. For the optimization of synthesis, several experiments were conducted. Consequently, various pH values (2, 6, and 8) were tested by adjusting the pH of the reaction mixture with HCl 0.1 M and NaOH 0.1 M. Afterward, several AgNO_3_ concentrations (1, 3, and 5 mM), extract and AgNO_3_ volume ratios (1:9, 5:5, and 9:1, *v*/*v*), temperatures (20, 40, and 60 °C), and reaction times (15, 30, 60, 90, and 120 min) were studied [52]. After optimization of AgNPs synthesis using E1 (AgNPs_1), the same conditions were used for AgNPs synthesis using E2 (AgNPs_2). The synthesized AgNPs were separated by centrifugation at 8000 rpm for 30 min, washed three times with distilled water in order to remove unwanted impurities, dried at 40 °C until constant mass, and stored until further use.

### 2.3. In Vitro Stability of AgNPs

The in vitro stability of AgNPs was investigated in different conditions and dispersion media. In total, 5 mL of colloidal dispersion was mixed with 5 mL of each medium, and the monitoring was made by recording the maximum absorbance initially and at different periods of time (7, 14, 21, and 28 days and 6 months). The media and conditions were represented by water (used as control), 5% NaCl solution, and phosphate-buffered saline solutions at pH 6, 7, and 8, at room temperature, kept in the dark.

### 2.4. Physicochemical Characterization of AgNPs

The formation of AgNPs was monitored by observing the change in the mixture color and by recording the UV–Vis spectra in the 300–600 nm range, using a Jasco V-530 UV–Vis double beam spectrophotometer (Tokyo, Japan).

FTIR spectroscopy was used to study the functional groups involved in synthesis, and the groups attached to the surface of AgNPs with capping and stabilizing roles. The analyses were performed on both the classic extract (E1) and its corresponding particles (AgNPs_1) and their spectra were recorded using a Bruker Vertex 70 spectrophotometer (Bruker, Billerica, MA, USA) in the 4000–310 cm^−1^ range.

The hydrodynamic diameter and the zeta potential were investigated by DLS analysis, using a Delsa Nano Submicron Particle Size Analyzer (Beckman Coulter Inc., Fullerton, CA, USA). Moreover, in order to obtain data on the size and morphology of AgNPs, TEM analysis was carried out, employing a Hitachi High-Tech HT 7700 microscope (Hitachi High-Technologies Corporation, Tokyo, Japan).

The elemental composition of AgNPs was determined by EDX analysis on an aluminum substrate using a Quanta 200 Environmental Scanning Electron Microscope (ESEM) with EDX (FEI Company, Brno, Czech Republic).

### 2.5. AgNPs Phytotoxicity on Triticum aestivum

The phytotoxicity test was performed according to Vannini et al. with some modifications [53]. The seeds of *T. aestivum* were immersed in 10% sodium hypochlorite solution for 10 min and then rinsed thoroughly with distilled water. After that, the seeds were left in water to inflate for 24 h. The inflated seeds were split and placed in Petri dishes, on filter paper impregnated with E1, AgNPs_1, E2, and AgNPs_2 (10 mg/L concentration) and water (control). The Petri dishes were kept at 24 °C and samples were added every day. On the 7th and 14th days, some plants were separated, and the roots and shoots were measured.

Regarding the microscopic analysis, the leaves of wheat seedlings collected after 14 days of treatment were stored in 70% ethanol. Transverse sections through the middle of the leaves were made with a hand microtome and were further analyzed using an Eclipse E400 microscope equipped with a Nikon D700 camera (Nikon, Tokyo, Japan).

### 2.6. Biological Activities

#### 2.6.1. Antimicrobial Testing

The antimicrobial activity of the samples was determined against three different reference strains: *Staphylococcus aureus* ATCC 25923, *Escherichia coli* ATCC 25922, and *Candida albicans* ATCC 10231. These reference strains were purchased from Mecconti (Warsaw, Poland). All microorganisms were stored at −80 °C in 20–40% glycerol. The bacterial strains were refreshed on nutrient agar (NA), and the yeast strain was refreshed on Sabouraud dextrose agar at 37 °C. MIC values were determined by a broth dilution method that was performed in 96-well microtiter plates using the resazurin reduction assay concept [54]. The bacterial culture grown to log phase was adjusted to 1 × 10^8^ cells/mL in nutrient broth. Inoculants of 50 μL were mixed with 50 μL of serial dilutions of samples and were subsequently incubated at 37 °C for 24 h. The preparation of resazurin involved dissolving it in a concentration of 0.015%, vortexing, filter-sterilizing through a 0.22 μm filter, and storing the end product at 4 °C for a maximum of two weeks. After plate incubation at 37 °C for 24 h, resazurin was added to all wells (20 μL per well) and further incubated for 2–4 h. MIC values were determined by reading the fluorescence at λ(EX) = 575 nm and λ(EM) = 590 nm with a FLUOstar Omega Microplate Reader (BMG LABTECH, Ortenberg, Germany). MIC was considered the lowest concentration of the antibacterial agent necessary to inhibit visible growth. MBC was determined by spreading 10 μL samples from wells on Plate count agar (PCA) plates. MBC was considered the lowest concentration that destroyed all bacterial cells. For the determination of MIC and MBC values, the experiments were performed in triplicate.

#### 2.6.2. Antioxidant Activity

Our study focused on the determination of antioxidant activity by ferrous ion chelating capacity, hydroxyl radical and DPPH radical scavenging assays, and lipoxygenase inhibition activity. Methods previously described in the literature were employed for all four tests [55,56,57,58,59,60]. In the ferrous ion chelating capacity assay, a pink complex with a maximum absorbance at 562 nm is formed by Fe^2+^ ions in the presence of ferrozine. The presence of a chelating agent in the reaction medium causes a reduction in the absorbance of the formed complex [61]. Regarding the hydroxyl radical scavenging activity, the hydroxyl radical formed in the reaction between the ferrous ion and hydrogen peroxide hydroxylates salicylic acid with the formation of a pink–purple compound with maximum absorbance at 562 nm [62]. For the third studied method, compounds with reducing potential neutralize the DPPH radical with a change in the color of the solution from violet to yellow and a decrease in the absorbance measured at 517 nm [63]. Moreover, active compounds can block 15-LOX by preventing the oxidation of linoleic acid and reducing the absorbance measured at 234 nm [64]. For those samples that presented ferrous ion chelating capacity, hydroxyl and DPPH radical scavenging activities, and LOX inhibition over 50%, EC_50_ values were calculated and expressed as μg sample/mL final solution. By using linear interpolation to determine the concentration of antioxidant agent corresponding to a 50% activity, the EC_50_ was determined by considering the first value that was below 50% and the first value that was above 50%, respectively.

## 3. Results

### 3.1. Optimization of AgNPs Synthesis

**Figure 1:**
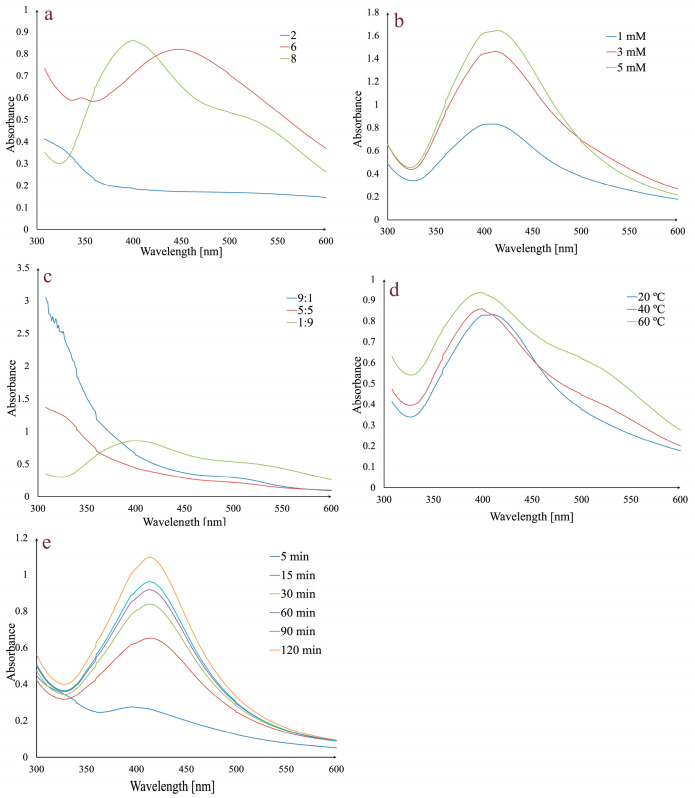
Effects of pH (**a**), AgNO_3_ concentration (**b**), extract:AgNO_3_ volume ratio (**c**) temperature (**d**), and reaction time on AgNPs synthesis (**e**).

Several variables, including pH, AgNO_3_ concentration, temperature, extract:AgNO_3_ volume ratio, and reaction time, were adjusted for the fastest and most effective synthesis of AgNPs. The reactions were monitored by UV–Vis spectroscopy in the 300–600 nm range (Figure 1).

In order to determine the influence of pH on AgNPs synthesis, different pH values were used (2, 6, and 8) (Figure 1a). No absorption peak was observed at pH 2, but for pH values 6 and 8, the presence of characteristic peaks in the 400–500 nm range indicates that the AgNPs synthesis has occurred. A pH value of 6 gave a broad peak at 450 nm, while at pH 8, the peak was higher, sharper, and shifted to a shorter wavelength (403 nm). Given that a shorter wavelength and a sharper peak indicate smaller AgNPs and a higher absorption peak indicates a higher concentration of AgNPs, pH 8 was selected for further analyses [65,66].

The effect of AgNO_3_ concentration on AgNPs synthesis was evaluated using 1 mM, 3 mM, and 5 mM solutions (Figure 1b). An increase in the absorption peak may be observed with the increase in theAgNO_3_ concentration. Given that the increase was negligible between 3 and 5 mM, the 3 mM concentration was chosen for synthesis.

Another studied parameter was represented by the extract:AgNO_3_ volume ratio. By analyzing Figure 1c, it can be observed that for 5:5 and 9:1 extract:AgNO_3_ volume ratios, the synthesis does not occur and only a 1:9 extract:AgNO_3_ volume ratio determines the synthesis. Consequently, the last value was chosen for further synthesis.

To evaluate the effect of temperature on AgNPs synthesis, several values (20 °C, 40 °C, and 60 °C) were taken into consideration (Figure 1d). The intensity of absorbance increases with temperature. At 20 °C, the absorption maximum was observed at 408 nm, while at 40 °C and 60 °C, it was shifted to 403 nm. Taking into consideration that at 60 °C the peak is broader, 40 °C was chosen as the optimal temperature value.

The last evaluated parameter was the reaction time (Figure 1e). For this, previously established conditions (1:9 extract:AgNO_3_ volume ratio, 3 mM AgNO_3_, pH 8, and 40 °C) were taken into consideration. The mixture was stirred, and the absorbance was monitored at different times in the 0–120 min range. After 5 min, a small absorbance peak was observed, which increased over time, with a maximum peak after 120 min, implying that silver ion reduction occurred and the AgNPs were synthesized.

In conclusion, the optimal experimental parameters for the synthesis of AgNPs were pH 8, 3 mM AgNO_3_ concentration, extract:AgNO_3_ volume ratio of 1:9, a temperature of 40 °C, and a reaction time of 120 min.

### 3.2. In Vitro Stability of AgNPs

**Figure 2:**
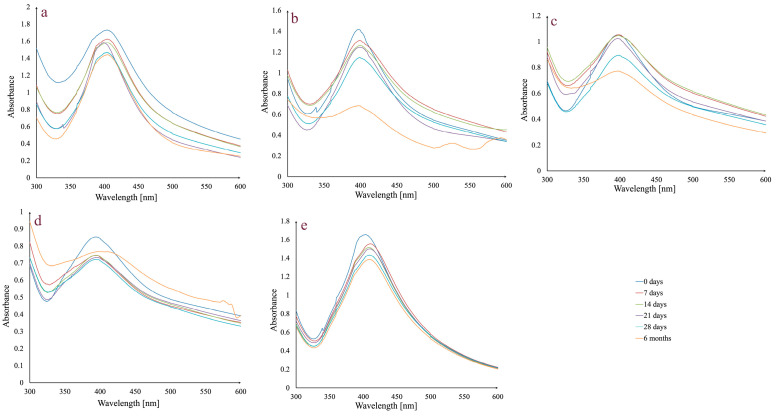
Absorbance variation in AgNPs_1 at different times, in different dispersion media: 5% NaCl solution (**a**); phosphate-buffered saline solution, pH 6 (**b**); phosphate-buffered saline solution, pH 7 (**c**); phosphate-buffered saline solution, pH 8 (**d**); and water (**e**) at room temperature, in the dark.

One of the most important requirements for any possible future use of AgNPs in medical applications is to guarantee colloidal dispersion stability for a specific amount of time. Therefore, preliminary studies were carried out on AgNPs_1 that were examined in different physiological dispersion media (5% NaCl solution, phosphate-buffered saline solution at pH 6, 7, and 8), versus the control (water), kept at room temperature, in the dark, for a period of up to 6 months (Figure 2).

In general, stability is maintained up to 28 days, during which time the peak stays in the same wavelength range and does not significantly change its aspect. There is a slight decrease in absorbance intensity between day 0 and day 28. Regarding the assessment conducted after six months, in the case of the 5% NaCl solution, the peak remains unchanged, while in the case of the phosphate-buffered saline solution, media differences can be observed. For all three pH values, the peak at 6 months changes its aspect by broadening. The most important decrease in absorbance intensity was observed in the case of phosphate-buffered saline solution pH 6.

### 3.3. Physicochemical Characterization of AgNPs

Following the green synthesis and the assessment of stability in various media, different approaches were used to determine the types of compounds that participate in the synthesis, as well as the size, shape, and surface structure of AgNPs.

#### 3.3.1. FTIR Analysis

**Figure 3:**
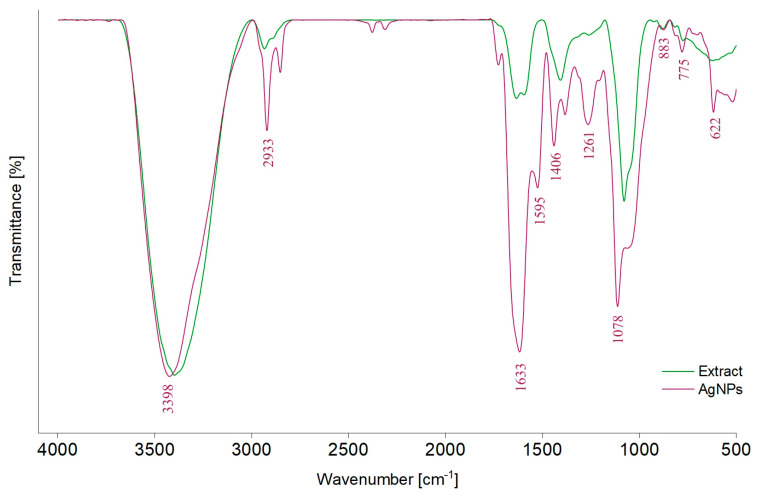
FTIR spectra of the *A. melanocarpa* classic extract (E1) and of the corresponding AgNPs (AgNPs_1).

FTIR analysis was used to identify the functional groups of phytocompounds that can participate during synthesis as capping and stabilizing agents (Figure 3).

In the FTIR spectrum of the classic extract (E1), different absorption bands can be observed: around 3398 cm^−1^, attributed to the stretching vibration of –OH groups (polyphenols, polysaccharides) [43,67,68], 2933 cm^−1^, corresponding to the stretching vibrations of C–H bonds from –CH, –CH_2_, and –CH_3_ (carbohydrates and sugars) [43,59,67,68,69], 1633 cm^−1^ and 1595 cm^−1^, belonging to C=O asymmetric stretching vibration [67], and 1406 cm^−1^ and 1261 cm^−1^, corresponding to phenolic C–O stretching of the pyran nucleus, typical for flavonoid C-rings [69]. Moreover, the band at 1600–1500 cm^−1^ can be attributed to the amide region of proteins [59]. The band at 1078 cm^−1^ corresponds to O–H variable angle vibration, 883 and 775 cm^−1^ to α- and β-glucosides, which were connected to form a pyranose ring [67] or C–H out-of-plane bending vibrations (B ring of flavonoids) [59] and the band at 622 cm^−1^ to C–H stretching of aromatic groups [70]. By comparing the values of absorption bands from the AgNPs_1 spectrum with those of E1, displacements can be observed.

#### 3.3.2. EDX Analysis

**Figure 4:**
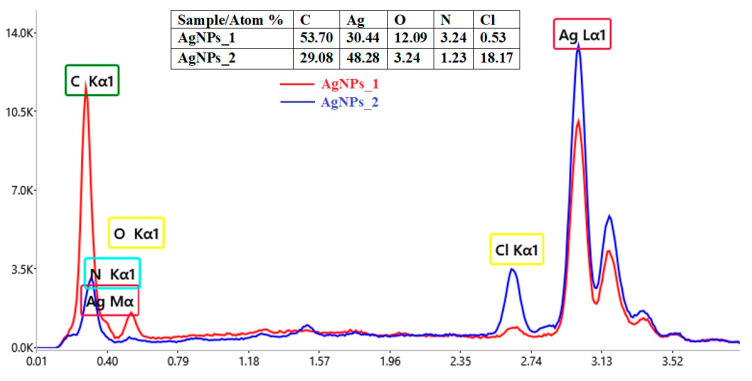
Comparative elemental composition of AgNPs obtained using *A. melanocarpa*.

Using EDX analysis, the elemental composition and atomic percentage of both types of biosynthesized AgNPs (AgNPs_1 and AgNPs_2) were determined (Figure 4).

The EDX spectra revealed the presence of a strong signal at 3 keV, for both AgNPs_1 and AgNPs_2. The results for other recorded signals, such as C, N, O and Cl, show a higher proportion of C atoms than those of Ag for AgNPs_1 (C/Ag ratio of 1.76), when compared to AgNPs_2 (C/Ag ratio of 0.6). However, in the case of the C/N ratio, the value for AgNPs_1 (C/N ratio of 16.57) is smaller than for AgNPs_2 (C/N ratio of 23.64). Moreover, a much higher percentage of Cl in AgNPs_2 (18.17%) compared to AgNPs_1 (0.53%) can easily be observed.

#### 3.3.3. DLS Analysis

**Figure 5:**
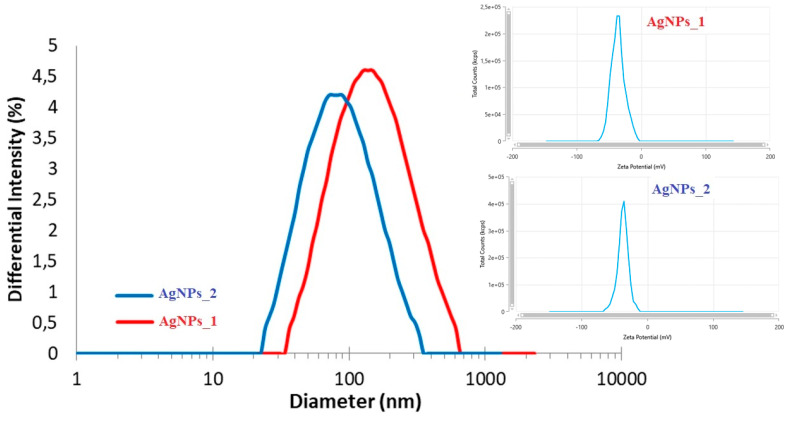
DLS analysis of AgNPs_1 and AgNPs_2.

In order to determine the hydrodynamic diameter and zeta potential of AgNPs, DLS analysis was used (Figure 5).

Even if the hydrodynamic diameter of AgNPs_1 (173.3 nm) was larger than that of AgNPs_2 (99.1 nm), the zeta potential had similar negative values in both cases (−35.96 mV for AgNPs_1 and −37.06 mV for AgNPs_2).

#### 3.3.4. STEM Analysis

**Figure 6:**
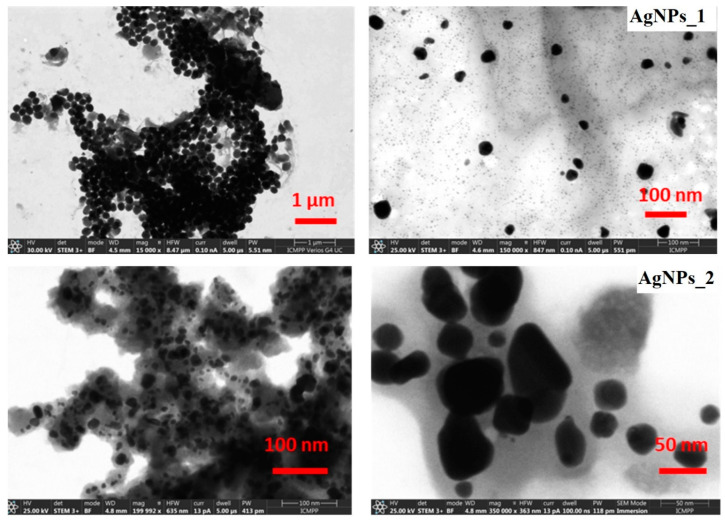
STEM micrographs at different magnifications of AgNPs_1 and AgNPs_2.

STEM microscopic analysis was used to study the size distribution and morphology of the AgNPs’ surface (Figure 6).

For AgNPs_1, a dimensional and morphological uniformity can be observed, with a diameter of approximately 40 nm and a spherical shape. The STEM micrographs of AgNPs_2 highlight that NPs have various sizes, ranging from 20 to 60 nm. Moreover, AgNPs_2 appear to have heterogeneous morphology, with spherical shapes at low dimensions up to around 30 nm and ovoid shapes at higher dimensions.

### 3.4. AgNPs Phytotoxicity on Triticum aestivum

**Figure 7:**
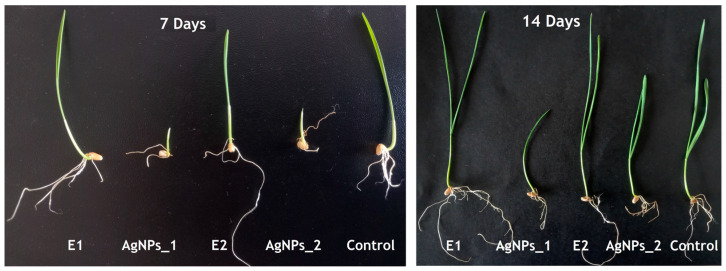
Effect of AgNPs on wheat growth after 7 and 14 days.

**Figure 8:**
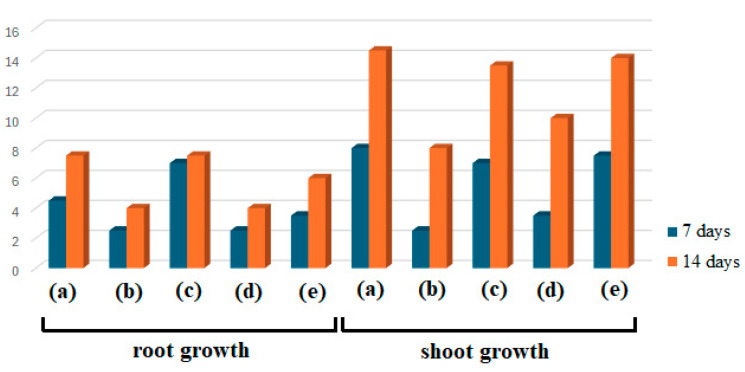
The root and shoot growth (in cm) of wheat seedlings treated with E1 (a), AgNPs_1 (b), E2 (c), AgNPs_2 (d), and control (e) at 7 and 14 days.

**Figure 9:**
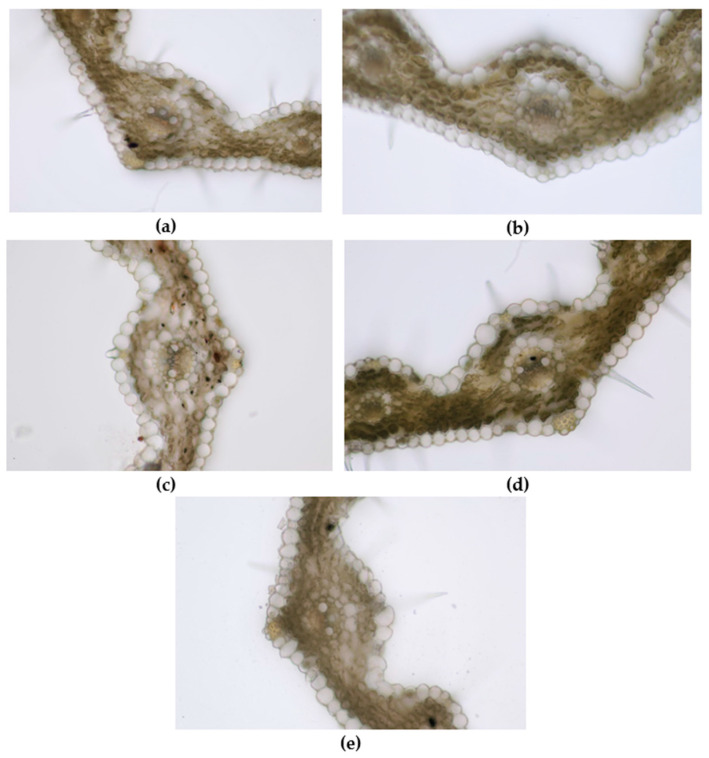
Leaf of wheat seedling treated with E1 (**a**), AgNPs_1 (**b**), E2 (**c**), AgNPs_2 (**d**), and control (**e**) (200×).

The first method used to evaluate the preliminary phytotoxicity on *Triticum aestivum* measured the growth of roots and shoots (Figure 7 and Figure 8). The second method involved microscopic examination of the leaves of wheat seedlings (Figure 9) treated with the two studied extracts (E1, E2) and their corresponding AgNPs, compared to the control (water).

As shown in Figure 7 and Figure 8, several differences between samples after 7 and 14 days can be noticed in both cases, considering root and shoot growth. Plants treated with AgNPs were found to have smaller roots than those treated with extracts or control, and their roots tended to point in the opposite direction as they avoided coming into contact with the filter paper impregnated with the studied samples, used as a seed germination substrate. Concerning dimensions, a similar finding was made regarding shoots. When comparing the sizes of the extract-treated and control shoots, E1 showed the highest growth, followed by control and E2. AgNPs_1 surpassed E2 when evaluating the plants’ growth after treatment with AgNPs.

Regarding microscopic analysis (Figure 9), the wheat grown on E1 had a well-developed leaf wider than the control and with numerous trichomes on both epidermises. The large vascular bundles present sclerenchyma sheaths and the same mechanical tissue is found above and below the bundle, just beneath the epidermises. The large bundles are fully developed, showing 2 big metaxylem vessels and 1–2 protoxylem elements with protoxylem lacuna. The small vascular bundles have incomplete parenchyma sheaths and only 2–3 cells of sclerenchyma under the upper epidermis. Small bundles have only slender metaxylem vessels. Abundant sclerenchyma tissue strengthens the margins of the leaf.

The leaf of wheat grown on AgNPs_1 was narrower than E1, with smaller and less developed vascular bundles. Although the mesophyll also had 3–4 rows of cells, the cells were smaller. The large vascular bundles had smaller metaxylem vessels than E1 and no protoxylem lacuna.

The leaf of wheat grown on E2 was comparable to that of E1 in terms of mesophyll size and development of vascular bundles but with significantly fewer trichomes on epidermises.

The wheat grown on AgNPs_2 showed well-developed vascular bundles and sclerenchyma reinforcements of vascular bundles. Two metaxylem vessels with a slightly smaller diameter than E2 were visible in the central vascular bundle. Furthermore, the leaf presented the highest number of trichomes and stomata on epidermises.

In the leaves of the control, the number of trichomes on both epidermises was significantly smaller than in E1 and the sclerenchyma sheath of the central vascular bundle was incomplete. Moreover, less mechanical tissue reinforced the veins. The mesophyll was narrower compared to E1 and the cuticle on the epidermises was thinner.

### 3.5. Biological Activities

#### 3.5.1. Antimicrobial Testing

**Figure 10:**
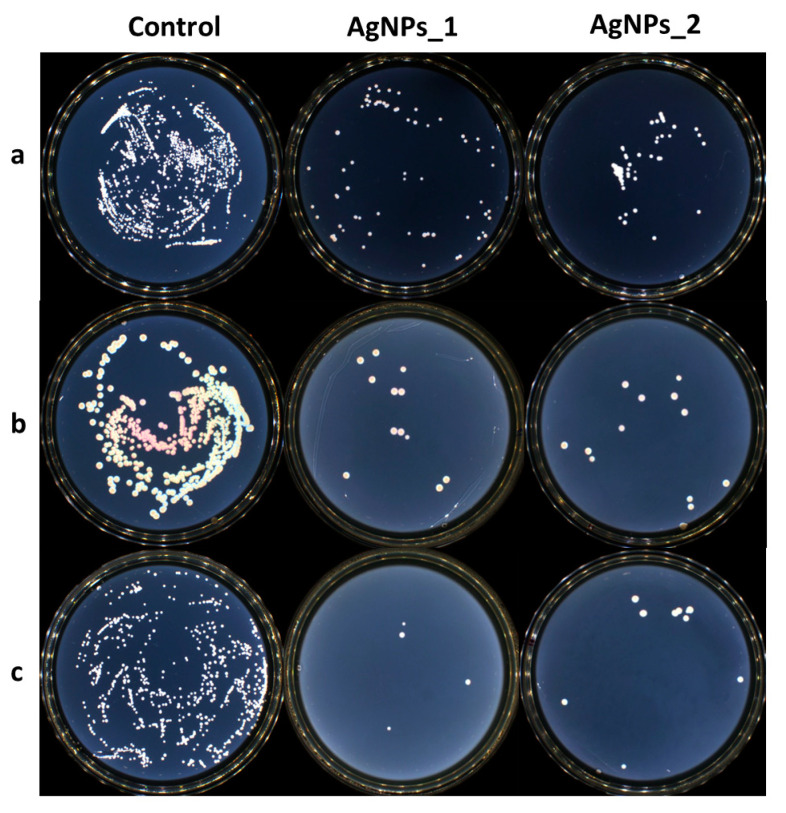
The growth inhibition of microorganisms for the control AgNPs_1 and AgNPs_2 against (**a**) *S. aureus*, (**b**) *E. coli*, and (**c**) *C. albicans*.

The antimicrobial activity of AgNPs was determined against three different reference strains, *S. aureus* ATCC 25923, *E. coli* ATCC 25922, and *C. albicans* ATCC 10231, using a broth dilution method. The growth inhibition of microorganisms can be seen in Figure 10.

Both tested samples presented antimicrobial activity against the studied strains. AgNPs_1 was efficient against *C. albicans*, with a MIC value of 0.312 mg/mL. AgNPs_2 proved to be more efficient with a MIC value of 0.156 mg/mL. At the same concentration of 0.625 mg/mL, AgNPs_1 decreased the visible growth of bacteria in both studied bacterial strains. AgNPs_2 had a minimum inhibitory concentration (MIC) of 1.250 mg/mL against *S. aureus* and 2.500 mg/mL against *E. coli*. The minimum bactericidal concentration (MBC) was not attained at the tested concentrations.

#### 3.5.2. Antioxidant Activity

**Figure 11:**
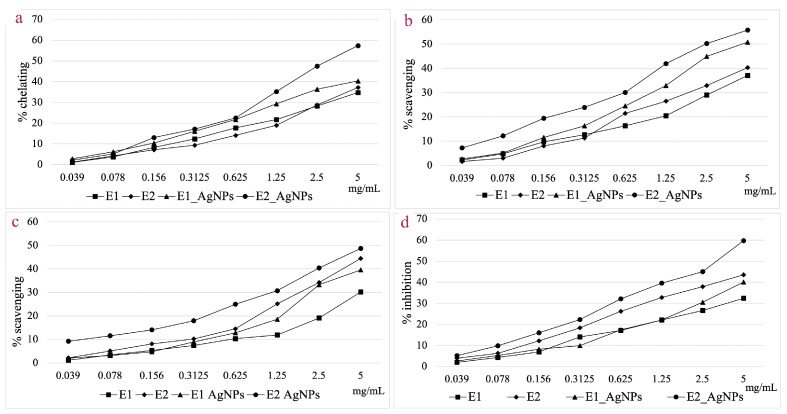
Ferrous ion chelating capacity (**a**); Hydroxyl radical scavenging activity (**b**); DPPH radical scavenging activity (**c**); In vitro LOX inhibition assay (**d**).

The current study focused on the determination of antioxidant activity by ferrous ion chelating capacity, hydroxyl radical and 2,2-diphenyl-1-picryl-hydrazyl (DPPH) radical scavenging, and lipoxygenase (LOX) inhibition assays. Figure 11 displays the comparative results obtained for E1, E2, AgNPs_1, and AgNPs_2 for each studied method.

When analyzing the ferrous ion chelating capacity at a concentration of 5 mg/mL, AgNPs_2 shows a 35.15% higher activity compared to E2, whereas for E1, the chelating capacity of AgNPs_1 is only 14.01% higher. For this assay, AgNPs_2 proved to be 29.67% more effective compared to AgNPs_1.

AgNPs showed a higher hydroxyl radical scavenging capacity compared to extracts at a concentration of 5 mg/mL. The efficiency compared to the corresponding extracts was 27.02% higher for AgNPs_1 and 27.61% higher for AgNPs_2, respectively. Both categories of NPs showed a scavenging capacity of over 50%, with the EC_50_ value for AgNPs_2 being 46.38% lower compared to that of AgNPs_1.

Generally, the hydroxyl radical scavenging activity is higher than that of DPPH radical scavenging for the tested samples. Considering the DPPH radical scavenging assay, the difference in efficiency of the AgNPs versus extracts was smaller when compared to the hydroxyl radical scavenging assay. This is particularly noticeable in the case of E2, where the increase in efficiency for AgNPs_2 was just 6.70% higher. Comparing the scavenging effect of the two types of particles, AgNPs_2 was 18.73% more effective compared to AgNPs_1, at a concentration of 5 mg/mL.

The LOX inhibition capacity was 19.23% higher in the case of AgNPs_1 compared to E1, and 27.09% higher for AgNPs_2 compared to E2.

## 4. Discussion

Since AgNPs possess free electrons in the conduction band, a phenomenon known as surface plasmon resonance (SPR) causes electrons on the metal silver surface to vibrate at particular wavelengths, resulting in a distinctive optical UV–Vis spectrum [71]. SPR is influenced by the size, shape, morphology, dielectric environment, and composition of AgNPs [66].

The SPR was first observed by a change in the mixture (extract—AgNO_3_ solution) color from yellow-reddish to dark brown, and afterward, by examining the UV–Vis spectra, taking into consideration different reaction conditions (pH, AgNO_3_ concentration, temperature, extract:AgNO_3_ volume ratio, and reaction time).

UV–Vis spectra can give important information regarding the size, shape, and stability of AgNPs. For example, the presence of a broad peak can indicate the tendency of AgNPs to aggregate, as well as a wide range of their size [71,72]. In this case, acidic conditions were not suitable for AgNPs synthesis, which can probably be linked to the inactivation of reducing agents from the extract [73]. Moreover, at pH 6, besides the broad peak observed, a very small secondary peak appears at shorter wavelengths that can be explained by the existence of quadrupole resonance in addition to the primary dipole resonance [74]. However, alkaline conditions proved favorable for synthesis. As the pH increases, the surface of the AgNPs becomes more negatively charged due to a higher degree of deprotonation of their functional groups. This negative charge causes electrostatic repulsion between the particles. The narrow peak at pH 8 indicates that the nanoparticles are monodisperse, have small sizes, and do not tend to aggregate [65,75]. This affirmation is supported by Mahiuddin et al. who, in the case of AgNPs synthesized using a *Piper chaba* stem extract, noticed that a low pH induced aggregation, while a higher pH favored the nucleation process and implicitly the synthesis of particles [76]. Alkaline pH values were also found to be optimal for AgNPs synthesized using extracts of *Vachellia tortilis* subsp. raddiana [77] or *Spinacia oleracea* [78].

An increase in the AgNO_3_ concentration from 1 mM to 3 mM determined an increase in absorbance and implicitly, a higher concentration of particles. However, the 3 mM and 5 mM concentrations showed similar values of absorbances.

The effect of temperature in the synthesis of AgNPs has been studied in the 20–60 °C range. An increase in temperature led to an increase in absorbance, which could indicate an increase in the amount of synthesized AgNPs. However, the broadening of the peak at 60 °C may indicate that a higher concentration in NPs may favor aggregation and an increase in their size implicitly [79,80]. Moreover, peak broadening can reveal the polydispersity of the AgNPs colloidal solution [81]. In our case, the optimum synthesis temperature was 40 °C. The same pattern was observed by Tesfaye et al. who investigated the synthesis of AgNPs using a *Vernonia amygdalina* extract in the 30–60 °C range. The intensity of the absorbance increases up to 50 °C and decreases at 60 °C. Initially, with increasing temperature, the kinetic energy of molecules increases, and silver ions are consumed faster, with the possibility of particle agglomeration being lower. However, the decrease in absorbance at 60 °C can be explained either by agglomeration of NPs or degradation of components on the surface of particles [82]. In this context, Akowuah et al. showed that 40 °C or 50 °C provides the highest polyphenol extraction and stability, while temperatures above 60 °C can cause the degradation of phenolic components [83].

The beginning of the Ag^+^ to Ag^0^ conversion was observed after 5 min by tracking the absorbance at various time intervals; nevertheless, this conversion occurs slowly, with the peak broadening. In time, the SPR band narrows and intensifies with a significant amount of Ag^+^ being converted. Therefore, a large number of AgNPs was obtained after 120 min. According to Dada et al., the optimum time reaction is reached when the SPR becomes narrow, as in the case of NPs synthesized using *Tithonia diversifolia*, for which the required time was 90 min [84].

The location of the SPR band on the UV–Vis spectra can explain the shape of AgNPs. In our case, the SPR band is positioned at 403 nm, and the formed AgNPs are spherical in shape [85,86].

Considering stability, the presence of a broad peak or a secondary peak in the UV–Vis spectra can indicate not only the presence of aggregates, but also a destabilization of AgNPs suspension [71]. In our case, measurements were performed in 5% NaCl solution and in phosphate-buffered saline solution with different pH values, which is significant since it mimics the ion concentration and the osmolality of the human body. The tested AgNPs exhibit stability in all studied media over a period of 28 days, with only a slight decrease in absorbance value. The most significant degradation appears after 6 months at pH 6, with a decrease and broadening of the main peak and the appearance of a secondary peak. This is consistent with the findings of Fernando et al. from long-term pH impact research, which showed that pH values between 5 and 7 corresponded to the most significant changes in AgNPs stability [87]. At pH 8, there was no decrease in the absorbance value, but there was a noticeable large peak with a red shift that suggests the existence of aggregates. It is likely that AgNPs undergo an oxidative dissolution at pH 6, where oxidized Ag^0^ interacts with protons to form Ag^+^, which is then released into the solution. However, at pH 7 and 8, the dissolution of Ag^+^ decreases over time [87]. Moreover, with increasing pH, organic functional groups from the AgNPs surface are deprotonated and the negative charge increases, which leads to an enhancement in the electrostatic repulsion and, implicitly, to smaller aggregates. The phytocompounds found on the surface of NPs limit excessive aggregation. This can also be observed when testing stability in 5% NaCl solution, where Na^+^ can protect the surface of AgNPs, ensuring electrostatic stabilization [88].

FTIR and chromatographic analyses were performed solely on the classic extract (E1) as supplementary research since it has a far more complicated chemical composition than the selective one (E2), which has already been previously characterized [51].

The FTIR spectrum of the classic extract revealed the presence of typical phytocompounds that can contribute to the reduction of Ag^+^ in Ag^0^, such as polyphenols, polysaccharides, and amino acids. When the FTIR spectra of AgNPs_1 and E1 are compared, similar peaks appear but in different positions, which could be correlated with the role of secondary metabolites in the stabilization and coating of particles.

In order to check the phytocompounds that could be responsible for the synthesis of AgNPs, an additional chromatographic study was performed on the classic extract (EI). The obtained results provide evidence in favor of the involvement of secondary metabolites in the generation of particles. Therefore, by screening 29 compounds, chlorogenic acid (101.487 μg/mL), 4-O-caffeoylquinic acid (26.422 μg/mL), hyperoside (4.135 μg/mL), isoquercitrin (4.357 μg/mL), rutoside (4.123 μg/mL), quercetin (0.504 μg/mL), gallic acid (0.87 μg/mL), and protocatechuic acid (18.46 μg/mL) could also be quantified in *A. melanocarpa* extract. Gentisic and caffeic acids were below the quantification limit. When analyzing the supernatant obtained after removing AgNPs, a decrease in the concentration of chlorogenic acid (5.384 μg/mL), 4-O-caffeoylquinic acid (1.779 μg/mL), and protocatechuic acid (2.81 μg/mL) was observed. Moreover, hyperoside, isoquercitrin, rutoside, and gentisic acid were below the quantification limit, and caffeic acid, quercetin, and gallic acid were no longer detected. Consequently, these compounds are to be considered examples of polyphenols that participate in the synthesis of NPs. Moreover, for both extract and supernatant, the total polyphenolic content was evaluated, and a decrease in the case of the supernatant (1.0434 mg GAE /mL to 0.1598 mg GAE/mL) was observed. Implicitly, polyphenols are a class of compounds that are responsible for the synthesis, capping, and stabilization of AgNPs.

According to the literature data, the high bioactivity of *A. melanocarpa* is due to polyphenols such as anthocyanins (cyanidine 3-glucoside, 3-galactoside, 3-xyloside and 3-arabinoside, pelargonidine-3-galactoside, and pelargonidine-3-arabinoside), phenolic acids (chlorogenic and neochlorogenic acids, cryptochlorogenic acid, *p*-coumaric acid and its derivatives, caffeic acid and its derivatives, protocatechuic, vanillic, ferulic, salicylic, syringic, 4-hydroxybenzoic, and ellagic acids), flavanols and flavonols (quercetin-3-glucoside, 3-galactoside, 3-rutinoside, 3-robinobioside and 3-vicianoside, isorhamnetin 3-galactoside, 3-glucoside, 3-neohesperidoside and 3-rutinoside, myricetin and kaempferol 3-galactoside, and 3-glucoside), and proanthocyanidins ((−)-epicatechin and (+)-catechin). Other classes of compounds that can participate during synthesis are amino acids (arginine, α-alanine, asparagine, cysteine, glutamic acid, histidine, lysine, serine, tyrosine, and threonine) or saccharides (fructose, glucose, sucrose, or sorbitol) [17].

The confirmation of AgNPs synthesis can be revealed through the presence of the characteristic absorption peak for silver in the EDX spectra. The other identified elements belong to phytocompounds present on the surface of AgNPs, which serve as capping organic agents [89]. The different C/Ag and C/N ratios for AgNPs_1 and AgNPs_2 suggest that the biomolecules attached to the surface of AgNPs differ in terms of both quality and quantity, with AgNPs_1 containing a higher concentration of biomolecules. Regarding the number of chlorine atoms, even though the conditions for synthesizing AgNPs were the same, the plant material and its processing differed, so the results could be explained by residual phytoconstituents from the extract or contaminants introduced into the sample during preparation [90,91].

The negative and high zeta potential values suggest a high stability of the colloidal solutions, given that negatively charged functional groups of biomolecules on the surface of AgNPs cause a higher dispersity due to the electrostatic repulsion. The presence of phytocompounds on AgNPs’ surface was also revealed by FTIR and EDX results, and by the higher diameter obtained during DLS analysis compared to STEM analysis. The hydrodynamic diameter comprises the silver core, the biomolecules on the surface, and the layer’s hydration, supporting the last statement [70]. Therefore, the dimensions measured by this method were smaller since STEM analysis measures only the silver core [92].

The structure of leaves includes an upper epidermis, a lower epidermis, and the photosynthetic tissue (mesophyll) in which vascular bundles are embedded. Both epidermises presented protective trichomes (hairs) and stomata. In the upper epidermis, groups of larger cells called motor cells were visible in the furrows between the veins. The mesophyll is a homogenous spongy parenchyma made of uniformly shaped and sized cells. The mesophyll comprises 3–4 rows of large rounded cells filled with chloroplasts and separated by intercellular spaces. All samples presented 11 vascular bundles (closed collateral with an adaxial strand of xylem and an abaxial strand of phloem), corresponding to parallel veins, and forming ridges on the upper epidermis. The mesophyll cells that border the vascular bundles are uniformly sized, large, and chloroplast-free. Comparing E1 and AgNPs_1, the reduction in size of xylem vessels may be a protection mechanism in order to reduce the uptake of toxic compounds, i.e., AgNPs [93].

Less developed vascular bundles through which nutrients are transported explain the decreased mesophyll size. Additionally, in comparison to E1, there is less sclerenchyma supporting the veins and leaf margins. The leaf presents numerous hairs on both epidermises, but slightly less than E1. These findings are in accordance with the literature data regarding anatomical changes induced by stress in wheat plants. Akcin et al. showed that exposure to chromium decreased the thickness of the phloem, xylem, and mesophyll in wheat leaves. The stems of chromium-treated plants exhibited a decrease in the sclerenchyma thickness, which was also noted in the current research for AgNPs_1 [94]. Moreover, copper stress caused a reduction in the diameter of vascular bundles and a decrease in the thickness of both epidermises of wheat leaves [95].

Despite containing a higher quantity of silver, it appears that leaf growth and structure were less influenced in AgNPs_2 compared to AgNPs_1. The existence of anthocyanidins in significant concentration, which have a protective effect against stress caused by metals, could provide a possible explanation. Anthocyanins increase a plant’s resistance to metals by chelating metal ions, scavenging free radicals to boost antioxidant defense, and isolating or sequestering toxic metals or metalloids in the vacuoles of plant cells [96,97]. It has been shown that foliar application of blueberry anthocyanins mitigates cadmium oxidative damage in rice leaves, and also isolates and immobilizes cadmium in soluble and organelle fractions [98]. Furthermore, exogenous application of anthocyanins enhances arsenic tolerance, leading to improved plant growth [99]. Another study reported that an anthocyanin-enriched extract from red cabbage leaves protects *Egeria densa* plants exposed to cadmium and manganese against metal toxicity [100].

The better development of E1 leaves compared to the control may be explained by the fact that the *Aronia* crude extract acted as a biostimulant through its various active compounds that are able to induce morphological and biochemical changes in wheat. Recent data showed that aqueous extracts from *Phyllanthus emblica*, *Plumbago zeylanica*, and *Baccopa monnieri* were highly effective in increasing wheatgrass length and fresh biomass [101]. Similarly, Rehman et al. reported that a *Moringa oleifera* leaf extract improves wheat growth and productivity [102].

It is well known that AgNPs possess antimicrobial activity, being able to destroy Gram-negative and Gram-positive bacteria, including multidrug-resistant strains [103] by accumulating in the inner membrane, thus generating destabilization and damage, by increasing membrane permeability and inducing leakage of cellular content [104] or by releasing Ag^+^, which can interact with cellular components, thus altering metabolic pathways [105,106]. AgNPs can penetrate fungal cells and disrupt cellular integrity. Consequently, the synthesis of DNA and RNA is affected, and the quantities of hydroxyl and peroxide radicals rise. In addition, proteins and lipids are damaged. Oxidative stress, spore germination inhibition, and fungal cell death are all linked to these processes [107].

The differences in efficiency against bacteria and fungi can be explained by the distinct structure of the cell wall. Gram-positive bacteria contain a thick peptidoglycan layer, while Gram-negative bacteria have an outer lipopolysaccharide membrane and a thin peptidoglycan layer. On the other hand, the fungal cell wall contains mainly polysaccharides, as well as proteins and lipids [108,109,110].

Phenolic compounds found in the extract, especially anthocyanins, can display antimicrobial activity [111]. Such compounds compromise the integrity of the bacterial cell wall and cell membrane by binding directly to bacterial DNA and interfering with protein homeostasis [112]. The literature also mentions that *A. melanocarpa* extracts possess antibacterial properties against *Proteus mirabilis*, *Proteus vulgaris*, *Bacillus subtilis*, *Staphylococcus aureus*, *Klebsiella pneumoniae*, *Pseudomonas aeruginosa*, and *Escherichia coli*, and antifungal properties against *Candida albicans* and *Aspergillus niger* [113,114]. Therefore, the antibacterial activity for the studied samples could be explained by the interaction of AgNPs and their surface composition. The charge surface, size, and shape of AgNPs, in addition to the Ag^+^ release profile and stabilizing phytocompounds from the surface, all determine the antibacterial potential [81,115,116]. The existing literature indicates that smaller AgNPs are more efficient than larger ones, since they generate more Ag^+^. Nevertheless, Gibala et al. showed that larger AgNPs can have greater biocidal efficacy than smaller AgNPs, and our findings support this claim [116].

Cells use ferrous ions to synthesize ferroproteins, out of which the most significant is hemoglobin. Ferrous ions can contribute to the production of hydroxyl radicals, which have a pro-oxidant effect and can cause several cardiovascular and neurological conditions, as well as cancer [117,118]. By chelating ferrous ions, the availability of ions for Fenton reactions decreases, oxidation reactions are blocked, and local inflammatory phenomena are reduced [119]. Polyphenols can chelate ferrous ions by covalent and coordinative bonds [120]. These compounds can initiate the reduction of ferric ions to ferrous ions, which will be chelated, thus preventing their contribution to uncontrolled oxidation reactions at the cellular level [121]. The complex chelation process mechanism involves the ion being either retained on the AgNPs’ surface or interacting with the functional groups of phytocompounds involved in the synthesis. Consequently, the extracts’ qualitative and quantitative composition can have an impact on the AgNPs’ ability to chelate iron [122].

The hydroxyl radical, one of the most reactive oxygen species, is synthesized in vivo through Fenton and Haber–Weiss reactions. It initiates oxidation chain reactions that lead to structural damage to proteins, lipids, and nucleic acids. The oxidation of lipids can cause the formation of lipid peroxides, which will contribute to the propagation of the oxidation process. Similarly, disruption of the phospholipid structure in the cell membrane and organelles impacts the stability and functioning of the cell [123]. Polyphenols contain hydrogen donor groups that participate in the synthesis of AgNPs and that can also neutralize free radicals, such as the hydroxyl radical [124]. The ability to neutralize this radical depends on the number of available hydroxyl groups and the concentration of polyphenols in extracts and AgNPs. Given that the synthesis of the hydroxyl radical depends on the presence of the Fe^2+^ ion, phytocompounds that can neutralize the hydroxyl radical and chelate the ferrous ion display both antioxidant and prooxidant blocking activities, both of which were highlighted for the studied extracts and AgNPs. The research carried by Li et al. on different fractions from *Aronia* fruits showed a good capacity of both monoglycosylated flavones and anthocyanins to neutralize the hydroxyl radical [125]. Furthermore, the antioxidant effects of chokeberry fruit extracts are dependent on the content of polyphenols and cyanosides [51,126]. The antioxidant activity through the neutralization of free radicals explains the protective effects of chokeberry fruits and extracts in various pathological phenomena [126].

DPPH is an unstable synthesis radical, which can be stabilized by compounds containing proton and electron donor groups (e.g., polyphenols). The hydroxyl groups of phytocompounds are capable of donating protons and electrons and participate in the neutralization of the DPPH radical. These groups are also involved in the synthesis process of metal NPs, which could explain the lower neutralizing effect compared to other antioxidant tests. Studies on *Aronia* extracts showed that the DPPH radical scavenger capacity mainly depends on the content of anthocyanins, followed by proanthocyanins, flavonols, and polyphenolic acids [127]. The capacity to neutralize the DPPH radical depends on the extraction method. Repeated extraction with warm water [128] or with water under pressure [113] allows the obtainment of extracts with a hydroxyl radical scavenging capacity of approximately 50%. The DPPH radical and the hydroxyl radical scavenger capacities depend on the existence of polyphenols with a catechol-type structure in the extract [129]. The DPPH radical scavenger capacity also depends on the employed chokeberry variety. Thus, the differences between the analyzed samples could also be explained by the different varieties used to prepare the extracts for AgNPs synthesis. The study carried out by Jakobek et al. revealed that the antioxidant efficacy varied by even five times amongst varieties [130]. The type of plant material employed for analysis also influences the antiradical action of chokeberry extracts; extracts derived from whole fruits have a lower antioxidant capacity than extracts made from fruit peel, which is very rich in anthocyanins [131].

The most important lipoxygenases involved in the oxidation of unsaturated fatty acids are 5-, 12- and 15-lipoxygenases. They contain iron in their active center, which takes part in the oxidation reaction that the enzyme catalyzes as ferrous or ferric ion. Lipoxygenases control the synthesis of lipid peroxides, which are important in physiological processes but can also lead to pathological ones including cancer, diabetes, atherosclerosis, neurodegenerative diseases, and asthma when produced in excess [132,133]. The blocking or reducing of LOX activity causes a decrease in the synthesis of lipid peroxides with a reduction in the risk of associated pathological phenomena. Since the enzyme’s activity depends on iron’s involvement in oxidation–reduction processes, phytocompounds that can release protons and electrons during the reduction in Fe^3+^ to Fe^2+^ followed by subsequent chelation will have an impact on the enzyme’s capacity to function. Phytocompounds can affect an enzyme’s spatial structure or the structure of its active center in addition to the oxidation–reduction process in which iron participates. Polyphenols found in plant extracts and on the surface of the synthesized AgNPs fall under this category. Compared to other antioxidant tests, the difference in activity between the two types of AgNPs was higher in the LOX inhibition test, with AgNPs_2 displaying a 32.85% higher inhibition capacity compared to AgNPs_1. This difference could be explained by the complex interaction between NPs and enzymes.

In addition to the previously mentioned factors, the antioxidant action can also be influenced by fertilizers used in the culture, fruit maturity at harvest, fruit processing technique, environmental conditions where the plants were cultivated, storage conditions of fruits from harvesting to processing, solvents used to obtain the extracts, extraction conditions, and temperature-processing conditions [134,135,136]. E2 shows a slightly better antioxidant activity compared to E1, due to the greater number of OH groups available to neutralize DPPH and hydroxyl radicals, chelate iron, and block LOX. Generally, AgNPs have higher antioxidant activity compared to the corresponding extracts, and out of the studied nanoparticles, AgNPs_2 proved to be more efficient. Therefore, AgNPs could be used for developing therapeutic options with local antibacterial and antioxidant effects.

These findings show promise in terms of the antibacterial and antioxidant qualities of the investigated AgNPs. The research does have several limitations, though. In this regard, findings might not precisely reflect in vivo conditions, which could constitute the aim of more thorough research in the future. Furthermore, this work did not investigate the mechanisms underlying the observed effects of AgNPs, which is an additional step toward comprehending their potential use.

## 5. Conclusions

The current study reports a simple and eco-friendly method for the synthesis of AgNPs using two different aqueous extracts of *A. melanocarpa*. The phytocompounds found in extracts act as reducing, capping, and stabilizing agents, as demonstrated by FTIR, EDX, and DLS analyses. The stability of AgNPs in different media was established by registering the absorbances of AgNPs in time, the most significant degradation is observed after 6 months at pH 6. TEM analysis revealed dimensional and morphological variations between the obtained AgNPs. Moreover, the samples used for wheat seedlings influenced the growth and structure of leaves differently. The biosynthesized NPs also presented antimicrobial activity. Considering the antioxidant potential, AgNPs generally displayed better activity compared to the corresponding extracts, with the results differing depending on the employed assay. In conclusion, our findings support the need for additional research to fully understand these actions as well as other additional effects of the biosynthesized AgNPs.

## Data Availability

Data are contained within the article.
